# Prognostic Significance of Neutrophil to Lymphocyte Ratio, Lymphocyte to Monocyte Ratio, and Platelet to Lymphocyte Ratio in Patients with Nasopharyngeal Carcinoma

**DOI:** 10.1155/2017/3047802

**Published:** 2017-02-22

**Authors:** Aiying Lu, Haifeng Li, Yuming Zheng, Minzhong Tang, Jun Li, Huihui Wu, Weiming Zhong, Jianquan Gao, Ningjiang Ou, Yonglin Cai

**Affiliations:** ^1^Wuzhou Health System Key Laboratory for Nasopharyngeal Carcinoma Etiology and Molecular Mechanism, Wuzhou, Guangxi 543002, China; ^2^Department of Clinical Laboratory, Wuzhou Red Cross Hospital, Wuzhou, Guangxi 543002, China; ^3^Department of Radiation Oncology, Wuzhou Red Cross Hospital, Wuzhou, Guangxi 543002, China

## Abstract

The peripheral blood neutrophil to lymphocyte ratio (NLR), lymphocyte to monocyte ratio (LMR), and platelet to lymphocyte ratio (PLR) have been reported to correlate with the prognosis of many malignancies. This study evaluated the prognostic value of pretreatment NLR, LMR, and PLR in nasopharyngeal carcinoma (NPC). A retrospective analysis of clinical and pathological data of 140 NPC patients without distant metastasis during initial treatment was conducted to identify correlations between NLR, LMR, and PLR and clinicopathological features, overall survival, and progression-free survival. Cox proportional hazard regression analysis was used to reveal the independent factors affecting the prognosis of NPC patients. NLR was associated with T staging, N staging, and overall clinical stage grouping of the NPC patients (*P* < 0.05). NLR ≥ 2.28, LMR < 2.26, and PLR ≥ 174 were significantly associated with a relatively short overall survival (*P* < 0.05). In addition, NLR ≥ 2.28 was significantly associated with a relatively short progression-free survival (*P* < 0.05). Cox proportional hazard regression analysis showed that NLR was an independent prognostic factor in NPC. Pretreatment NLR, LMR, and PLR might be a useful complement to TNM staging in the prognostic assessment of NPC patients.

## 1. Introduction

Nasopharyngeal carcinoma (NPC) is one of the most common head and neck cancers in Southeast Asia, with a particularly high incidence in the provinces of Southern China [1–3]. Due to its anatomical location and radiosensitivity, radiotherapy or combined chemotherapy is a standard treatment for NPC. As for other solid tumors, the prognosis of NPC mainly depends on the TNM staging system [4]. However, TNM staging alone cannot predict NPC treatment efficacy. NPC patients with the same clinical staging often have different clinical courses. The possible explanation is that TNM staging is mainly based on the anatomical information and does not reflect the biological heterogeneity of the tumor. Hence, identification of prognosis-related biological markers may be an effective complement to TNM staging for the prognostic assessment of NPC patients.

Recent studies have shown that systemic inflammation promotes tumor progression and metastasis via the inhibition of apoptosis, promotion of angiogenesis, and damaging of DNA [5]. Hematological indices for these systemic inflammatory conditions, such as leukocyte count, monocyte count, platelet count, neutrophil to lymphocyte ratio (NLR), lymphocyte to monocyte ratio (LMR), and platelet to lymphocyte ratio (PLR), have been found to be independent prognostic factors for patients with non-small cell lung cancer [6], gastric cancer [7], and breast cancer [8]. This study evaluated the correlations between NLR, LMR, and PLR and clinicopathological features of NPC for the evaluation of their prognostic value in NPC patients.

## 2. Materials and Methods

### 2.1. Patients

In this study, 140 NPC patients admitted to Wuzhou Red Cross Hospital (Guangxi, China) from Feb 2009 to May 2010 were recruited, and the study protocol was approved by the Ethics Committee of Wuzhou Red Cross Hospital. The patients comprised 101 males and 39 females with a median age of 47 (range 10–76 years). The following criteria were applied for the inclusion of research subjects in this study: (1) pathologically diagnosed with NPC; (2) no prior malignancy; (3) no distant metastasis; (4) no current antitumor therapy; and (5) no infection or symptoms of inflammation. All patients were clinically staged in accordance with Chinese 2008 staging system [9] and received radical radiotherapy. Stages at III and IVa NPC patients received a combination of radiotherapy and chemotherapy.

### 2.2. Blood Tests

A blood sample was collected from each patient in an EDTA anticoagulant-treated tube and analyzed for routine peripheral blood cells (e.g., lymphocytes, neutrophils, monocytes, eosinophils, basophils, and platelets) using a Sysmex XE-2100 automated hematology system and its reagent kits (Sysmex, Japan).

### 2.3. Statistical Analysis

SPSS 13.0 (SPSS, Chicago, IL) software was used for statistical analysis in this study. Hematological indices were presented as medians (minimum to maximum value). In each patient, NLR was calculated by dividing the neutrophil number by the lymphocyte number; LMR was calculated by dividing the lymphocyte number by the mononuclear cell number; and PLR was calculated by dividing the platelet count by the lymphocyte number. A Chi-square (*χ*^2^) test was used to determine the correlations between NLR, LMR, and PLR and clinicopathological features of the NPC patients. ROC curve analyses were used to determine the best cut-off values of the hematological indices for patients' survival. A Kaplan-Meier analysis was used to calculate patients' survival and prepare survival curves. The log-rank test was used to compare the survival rate in each group. A Cox proportional hazard regression analysis was used to evaluate multiple prognostic factors. Overall survival (OS) was defined as the duration from diagnosis to death or last follow-up. Progression-free survival (PFS) was the duration from diagnosis to local recurrence/metastasis or last follow-up. *P* < 0.05 was considered as statistically significant.

## 3. Results

### 3.1. Patients' Survival

One hundred and forty NPC patients had completed treatment, including 34 cases (24.3%) of radiotherapy alone and 106 cases (75.7%) of combined radiotherapy and chemotherapy. Median follow-up of the patients was 68 months (range 5–77 months). Eleven patients were lost to follow-up; 17 patients had local recurrence; 21 patients had distant metastasis; and 29 patients died. Among the NPC patients, the 5-year OS was 78.8%, and the 5-year PFS was 76.2%.

### 3.2. Association between Pretreatment NLR, LMR, and PLR, and Clinicopathological Features of the NPC Patients

Of the 140 NPC patients, the medians of pretreatment peripheral blood lymphocytes, neutrophil number, monocyte number, platelet count, NLR, LMR, and PLR were 1.76 × 10^9^/L (0.52–3.61), 3.96 × 10^9^/L (1.47–10.86), 0.52 × 10^9^/L (0.01–2.00), 234 × 10^9^/L (61–370), 2.34 (0.70–6.60), 3.31 (0.89–320), and 136 (43–361), respectively.

ROC curves are constructed between death events and censors. The cut-off values of pretreatment NLR, LMR, and PLR were 2.28, 2.26, and 174, respectively, and were used to divide the NPC patients into high- and low-level groups. NLR was significantly correlated to the T staging, N staging, and overall clinical stage grouping of the NPC patients (*P* < 0.05) but did not correlate with the age, gender, and pathological type (*P* > 0.05). LMR and PLR were not significantly correlated to the age, gender, pathological type, T staging, N staging, or overall clinical stage grouping of the NPC patients (*P* > 0.05, Table 1).

### 3.3. Correlations between Pretreatment NLR, LMR, and PLR and Prognosis of the NPC Patients

**Figure 1:**
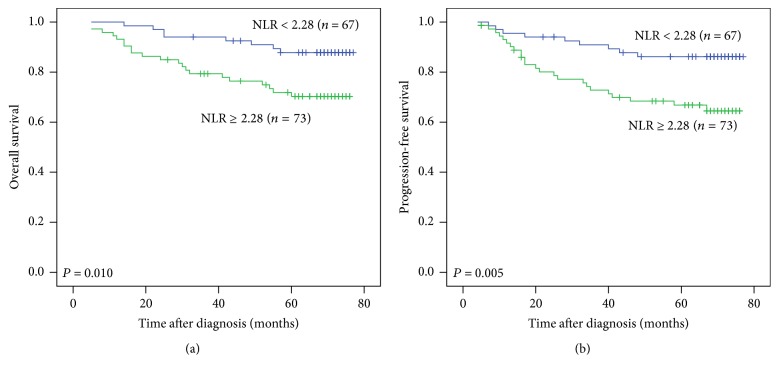
Kaplan-Meier curves for overall survival (OS) and progression-free survival (PFS) of patients with nasopharyngeal carcinoma according to pretreatment neutrophil to lymphocyte ratio (NLR). (a) OS stratified by NLR. (b) PFS stratified by NLR.

Five-year OS and 5-year PFS of the patients with pretreatment NLR ≥ 2.28 were significantly lower than the patients with pretreatment NLR < 2.28 (OS: 70.3% versus 87.8%, *P* = 0.010; PFS: 66.8% versus 86.2%, *P* = 0.005) (Figure 1).

**Figure 2:**
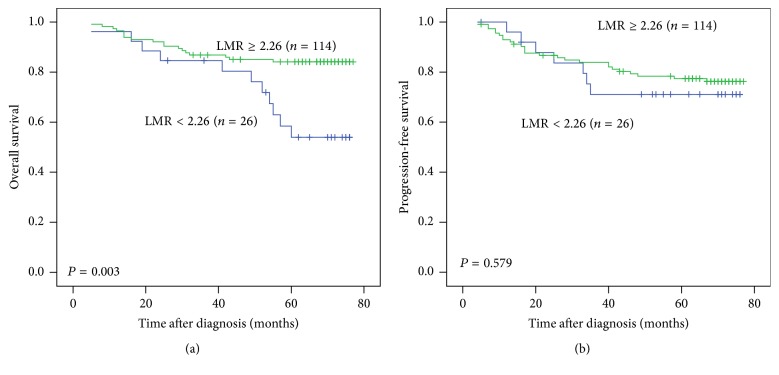
Kaplan-Meier curves for overall survival (OS) and progression-free survival (PFS) of patients with nasopharyngeal carcinoma according to pretreatment lymphocyte to monocyte ratio (LMR). (a) OS stratified by LMR. (b) PFS stratified by LMR.

As shown in Figure 2, 5-year OS of the patients with pretreatment LMR < 2.26 was significantly lower than the patients with pretreatment LMR ≥ 2.26 (53.9% versus 84.1%, *P* = 0.003). Five-year PFS of the patients with pretreatment LMR < 2.26 and ≥ 2.26 were 71.1% and 77.4%, respectively, which was not a significant difference (*P* = 0.579).

**Figure 3:**
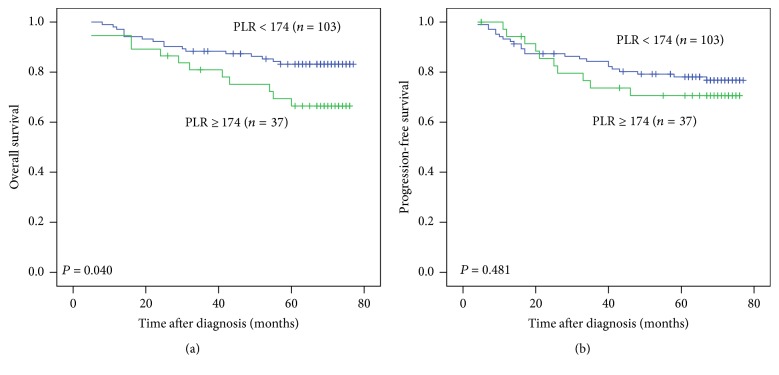
Kaplan-Meier curves for overall survival (OS) and progression-free survival (PFS) of patients with nasopharyngeal carcinoma according to pretreatment platelet to lymphocyte ratio (PLR). (a) OS stratified by PLR. (b) PFS stratified by PLR.

In Figure 3, 5-year OS of the patients with pretreatment PLR ≥ 174 was significantly lower than the patients with pretreatment PLR < 174 (66.5% versus 83.2%, *P* = 0.040). Five-year PFS of the patients with pretreatment PLR ≥ 174 and < 174 were 70.6% and 78.1%, respectively, which was not a significant difference (*P* = 0.481).

The associations of pretreatment NLR, LMR, and PLR with OS and PFS were examined further with Cox proportional hazards regression modeling, with adjustment for age, gender, WHO pathological classification, clinical staging, and chemotherapy. The results showed that NLR was an independent prognostic factor of OS and PFS in NPC patients; in addition, T staging was also an independent prognostic factor of OS (Table 2).

## 4. Discussion

Inflammation is commonly recognized to play a key role in cancer development and possibly affects the survival of cancer patients [10]. In this study, we found that patients with high T staging, N staging, and locally advanced NPC had relatively high pretreatment NLR. Increased NLR was significantly associated with poor OS and PFS of the patients, suggesting that NLR was independent prognostic factors for NPC. These results were consistent with the findings of An et al. [11]. The mechanism behind poor tumor prognosis due to an increase in NLR remains unclear. Neutrophils, as a type of inflammatory cells, are considered to be involved in different steps of tumor development through the production of a variety of cytokines, such as oncostatin M, hepatocyte growth factor, and transforming growth factor- (TGF-) *β* [12]. In addition, neutrophils promote tumor angiogenesis through the release of angiogenic factors, such as vascular endothelial growth factor, angiopoietin-1, and fibroblast growth factor-2 [13, 14]. On the other hand, lymphocytes are also responsible for immune surveillance to remove tumor cells. The involvement of lymphocytes, such as T cells, in tumor infiltration is associated with better prognosis of cancer patients and has been used for tumor-targeted therapy [15, 16]. Therefore, NLR may affect the tumor microenvironment and immune system to influence the survival of NPC patients.

Tumor-infiltrating mononuclear cells were found to promote tumor invasion and cancer cell growth in lymphoma [17]. In addition, an increased number of monocytes before treatment has been associated with poor prognosis of lymphoma and other solid tumors [18, 19]. The results of this study also showed that a relatively high LMR was associated with better survival in NPC. Monocytes release monocyte chemoattractant protein- (MCP-) 1 to stimulate and mediate tumor-associated monocyte infiltration in solid tumors and then produce various chemokines, such as TGF-*α*, tumor necrosis factor- (TNF-) *α*, interleukin- (IL-) 1, and IL-6 which promote tumorigenesis, angiogenesis, and distant metastasis of malignant tumors [20]. NPC is usually infiltrated with lymphocytes such as Th17 cells. Th17 cells are partly regulated by macrophage migration inhibitory factor (MIF) and could generate a high level of cytokines including TNF and interferon- (IFN-) *γ*, which mediate the antitumor effects [21]. In NPC, infiltrating lymphocytes, including Th17 cells, can express MIF, and a high level of MIF was associated with a better treatment outcome of the disease [22]. Monocytes are likely to play an opposite role of lymphocytes and promote tumor development in NPC.

Platelets not only are involved in blood coagulate functions but also secrete a variety of cytokines to directly or indirectly participate in the inflammatory responses of the body. Chen et al. [23] reported that pretreatment increased platelet count was an unfavorable prognostic factor for NPC patients. In this study, a relatively high PLR was associated with short survival in NPC patients. The impact of the interaction between tumor progression and platelets remains unclear. Sharma et al. [24] have reported that platelets could mediate tumor growth, proliferation, and angiogenesis. Activated platelets promote tumor cell growth and survival through paracrine signaling or direct contact and interaction with tumor cells.

NLR, LMR, and PLR, as the ratio of absolute counts between 2 types of cells, have relative stability. Elevations of NLR, LMR, and PLR are often due to an imbalance between the 2 types of cells, and it can be considered that the balance between tumor-promoting inflammatory and antitumor immune status is violated. Patients with elevated NLR or PLR and decreased LMR denote that the balance is tipped in favor of tumor-promoting inflammatory with promoting tumor cell proliferation and cancer metastasis while weakening anti-tumor protection and is associated with poor oncologic outcome. NLR, LMR, and PLR measurements are easily obtained in clinical practice from routine blood tests. Therefore, they may be useful complements to the overall assessment of the clinicopathology of NPC patients.

## 5. Conclusion

In the current study, our results indicate that the pretreatment NLR was an independent prognostic factor in NPC, and NLR, LMR, and PLR might be a useful complement to TNM staging in the prognostic assessment of NPC patients. Limitations of this study include its small sample size and single-centered retrospective design. Multicenter, large scale prospective studies will be necessary to define the precise cut-off values of NLR, LMR, and PLR as prognostic markers for NPC.

## Figures and Tables

**Table 1:** Association of pretreatment NLR, LMR, and PLR with clinicopathologic characteristics in patients with nasopharyngeal carcinoma.

Characteristics	Case	NLR			LMR			PLR		
		<2.28	≥2.28	*P*	<2.26	≥2.26	*P*	<174	≥174	*P*
Age (year)										
<45	55	28	27	0.561	10	45	0.924	41	14	0.833
≥45	85	39	46		16	69		62	23	
Sex										
Male	101	47	54	0.614	21	80	0.277	76	25	0.469
Female	39	20	19		5	34		27	12	
Pathology (WHO)										
Type I/II	23	11	12	0.997	5	18	0.669	16	7	0.634
Type III	117	56	61		21	96		87	30	
T classification										
T_1_-T_2_	63	36	27	0.047	8	55	0.106	49	14	0.307
T_3_-T_4_	77	31	46		18	59		54	23	
N classification										
N_0_-N_1_	43	26	17	0.047	8	35	0.995	33	10	0.571
N_2_-N_3_	97	41	56		18	79		70	27	
Overall stage										
I-II	19	14	5	0.015	2	17	0.332	15	4	0.568
III-IVa	121	53	68		24	97		88	33	

NLR: neutrophil to lymphocyte ratio; LMR: lymphocyte to monocyte ratio; PLR: platelet to lymphocyte ratio; Type I: keratinizing squamous cell carcinoma; Type II: differentiated nonkeratinizing carcinoma; Type III: undifferentiated nonkeratinizing carcinoma.

**Table 2:** Multivariate analysis of prognostic factors for survival of NPC patients using Cox proportional hazards regression model.

Endpoint	Hazard ratio (95% CI)	*P*
Overall survival		
T classification: T_3-4_ versus T_1-2_	3.500 (1.387–8.830)	0.008
NLR (≥2.28 versus <2.28)	2.383 (1.041–5.457)	0.040
Progression-free survival		
NLR (≥2.28 versus <2.28)	2.615 (1.206–5.672)	0.015

NPC: nasopharyngeal carcinoma; NLR: neutrophil to lymphocyte ratio; CI: confidence interval.
